# Successful treatment of diffuse alveolar hemorrhage secondary to *Mycoplasma pneumoniae* complicated with hemophagocytic lymphohistiocytosis in children: a case report and non-systematic literature review

**DOI:** 10.3389/fped.2024.1404872

**Published:** 2024-07-10

**Authors:** Min Yang, Zhong-Qiang Liu, Yang Wang, Li-Li Luo, Li-Na Qiao

**Affiliations:** ^1^ Department of Pediatrics, West China Second University Hospital, Sichuan University, Chengdu, China; ^2^ Key Laboratory of Birth Defects and Related Diseases of Women and Children of Sichuan University, Ministry of Education, Chengdu, Sichuan, China; ^3^ NHC Key Laboratory of Chronobiology, Sichuan University, Chengdu, China

**Keywords:** diffuse alveolar hemorrhage, hemophagocytic lymphohistiocytosis, *Mycoplasma pneumoniae*, children, case report

## Abstract

**Background:**

After quarantine-related measures were completely lifted in China, the respiratory infection rate of children caused by *Mycoplasma pneumoniae* (MP) increased significantly, and MP infection may lead to rare severe intra- and extrapulmonary manifestation. Hemophagocytic lymphohistiocytosis (HLH) and diffuse alveolar hemorrhage (DAH) are life-threatening clinical syndromes. Timely recognition may contribute to timely treatment and an improved prognosis. Currently there are no reports of children with DAH secondary to MP infection complicated with HLH.

**Case presentation:**

We successfully treated a previously healthy school-aged child who was admitted to the pediatric intensive care unit with fever, cough, drowsiness, and progressive dyspnea. HLH was confirmed by clinical and testing criteria, DAH was indicated by computed tomography scan of the chest, and *Mycoplasma* antibody detection and endotracheal aspirates pathogen metagenomic next-generation sequencing (mNGS) confirmed MP infection. After invasive mechanical ventilation, antibiotics, and glucocorticoid treatment, the patient recovered well and was discharged. At follow-up, she did not experience any more initial symptoms. For the fourth consecutive month, all indexes remained normal.

**Conclusion:**

mNGS can be considered for identifying the causative agent of infection in patients with DAH and/or HLH. The clinical manifestations of DAH in children may only present as acute hypoxic respiratory failure, significantly decreased hemoglobin without bleeding elsewhere, and chest imaging findings may assist in the diagnosis of DAH. When MP infection is associated with hemocytopenia, HLH should be considered.

## Introduction

*Mycoplasma pneumoniae* (MP) is a major pathogen of respiratory infection in school-aged children in China, which can lead to abnormal clinical intra- and extrapulmonary manifestation through direct injury and abnormal immune response of the host. As a potentially fatal disease, MP-hemophagocytic lymphohistiocytosis (HLH) is a form of related hematological manifestation induced by MP through immune dysregulation. Only a few cases were reported before the global quarantine-related measures were lifted (1–4). After the quarantine-related measures were lifted, no relevant reports were currently reported through PubMed, Web of Science, Embase, Medline, and other databases. Diffuse alveolar hemorrhage (DAH) is a potentially life-threatening syndrome with a poor overall prognosis (5). Through the above database search, only one case of DAH secondary to MP has been reported to date (6).

Here, we reported a rare case of DAH secondary to MP-HLH in children. In addition, the literature in this paper was also reviewed to provide clues for early identification of rare severe intra- and extrapulmonary manifestation, so as to timely intervene and improve the prognosis.

## Case presentation

A girl, aged 9 years and 5 months, was admitted to the Pediatric Intensive Care Unit (PICU) of West China Second Hospital of Sichuan University in November 2023. Her chief complaint was “fever for 5 days, cough and drowsiness for 3 days, and shortness of breath for half a day.” Only fever with no other symptoms occurred on the first and second days, with the highest temperature of 38.5°C (101.3°F) on day 1 and 39.5°C (103.1°F) on day 2, and was treated with oral antipyresants. The fever persisted, and cough and drowsiness occurred on day 3. After emergency treatment with cefoperazone sodium sulbactam for anti-infection, glutathione for liver protection, and gamma globulin (12.5 g, 0.5 g/kg) support on days 4 and 5, there was no improvement, and shortness of breath (56 breaths/min) occurred on day 5. The patient was previously healthy without family history, and no family members had a similar medical history. On admission, physical examination revealed poor mental response and slight irritability, a respiratory rate of 53 breaths/min, heart rate of 134 beats/min, pulse oxygen saturation (SPO_2_) of 90% without oxygen inhalation, slight edema of bilateral eyelids, nares flaring, three depression sign was positive, no rales heard in both lungs, and no abnormalities found in physical examination of the heart, abdomen, and nervous system. A routine blood test showed thrombocytopenia [platelets (PLT) 69,000/μl] and anemia [hemoglobin (Hb) 9.8 g/dl]. The decrease was 1.9 g/dl within 24 h, white blood cells (WBC) were decreased (3,500/μl, neutrophil count 1,870/μl). Serum C-reactive protein (CRP) was 119.8 mg/L, liver enzymes were elevated [aspartate aminotransferase (AST) 241 U/L; alanine aminotransferase (ALT) 372 U/L], lactate dehydrogenase triglyceride (LDH) was 1378 IU/L, triglyceride (TG) was 3.01 mmol/L, and coagulation function screening was negative. A pharyngeal swab of molecular diagnostic testing was negative for MP. The patient was initially diagnosed with severe pneumonia with respiratory failure and suspected HLH.

**Figure 1:**
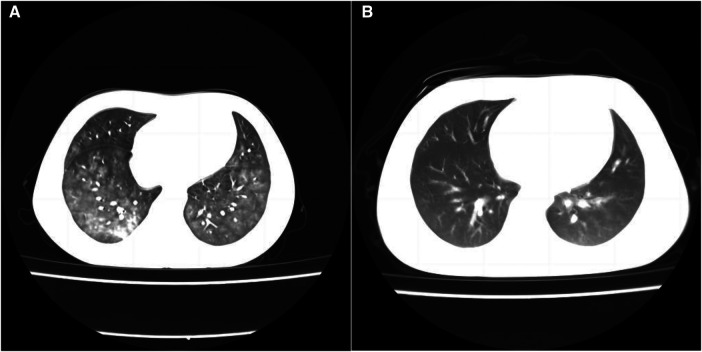
Chest CT scanning of the patient. CT lung window on admission showed diffuse small ground-glass opacities in both lungs with patchy opacities in the right lower lobe, and diffuse alveolar hemorrhage was considered (**A**). The CT lung window on the 11th day after admission showed a little inflammation in both lungs, and the lesions in both lungs were significantly reduced and faded (**B**).

**Figure 2:**
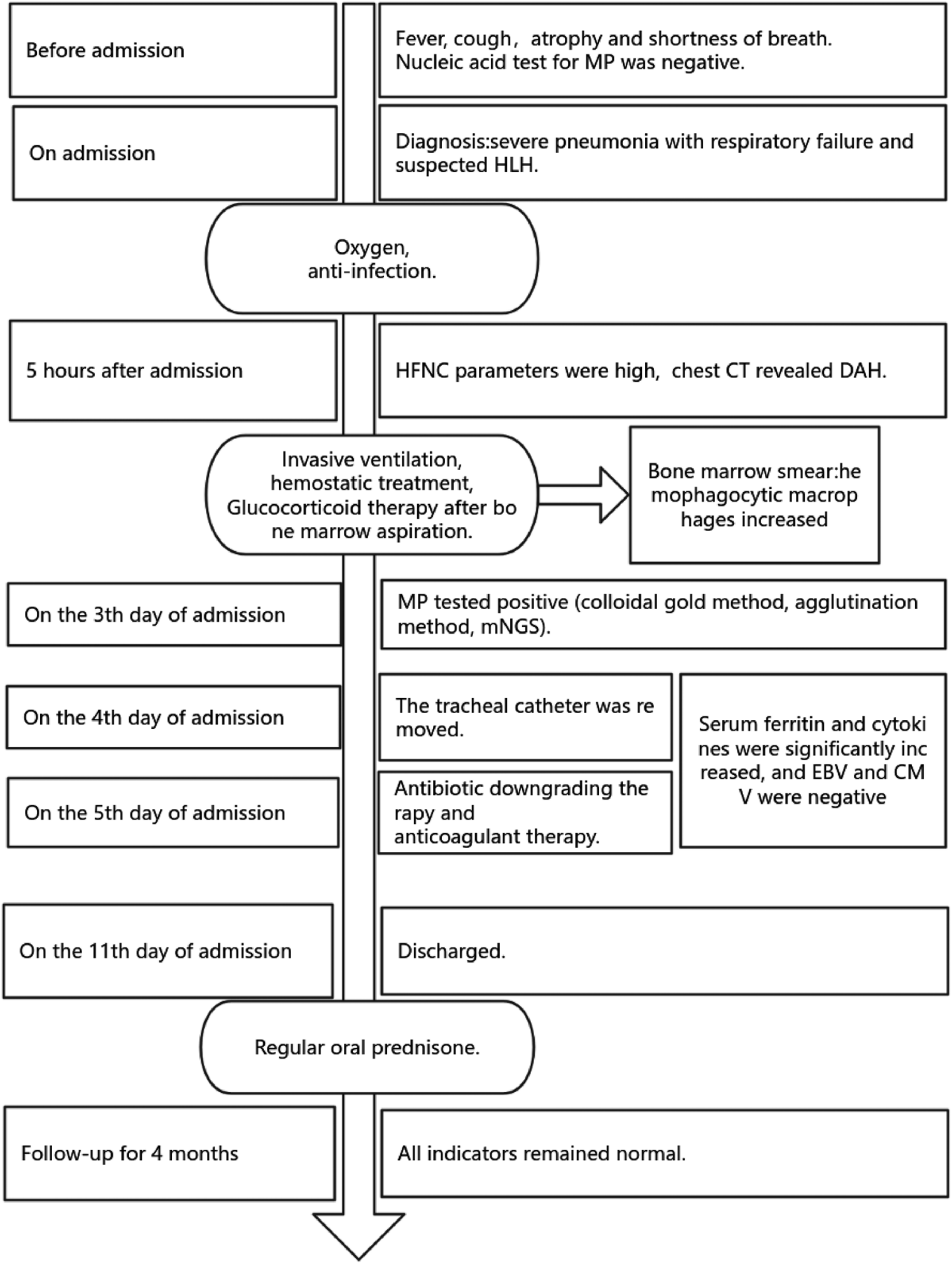
The timeline of disease progression and treatment.

Upon admission, the patient received a high-flow nasal cannula (HFNC) for assisted ventilation, meropenem (40 mg/kg, q8h, for 5 days) combined with oral azithromycin (10 mg/kg, qd, for 5 days, two courses) empiric antibiotic treatment. Five hours after admission, the patient's dyspnea did not improve with the HFNC parameters (FiO_2_ 60%, flow 8 L/min) and chest computed tomography (CT) revealed diffuse alveolar hemorrhage (Figure 1A). We used invasive ventilator-assisted ventilation after tracheal intubation (a few hemorrhagic substances were visible in the tracheal tube without bleeding in other parts), hemostatic treatment with plasma, ethylsulfonamide and platelet transfusion, and glucocorticoid therapy [methylprednisolone, intravenously guttae (ivgtt), 2 mg/kg/day for 5 days, 1 mg/kg/day, ivgtt, for 5 days; after that, oral prednisone was gradually reduced and discontinued] after bone marrow aspiration. After the treatment, the dyspnea was relieved (respiratory rate of 29 breaths/min, SPO_2_ 98%, no nares flaring, three depression sign was negative) and no hemorrhagic substance was visible in the tracheal tube. On the third day after admission, the MP antibody IgM (colloidal gold method, agglutination method) was positive, and the titer was >1:1280. Metagenomic next-generation sequencing (mNGS) of endotracheal aspirates was MP (amplified sequence number 98493, high confidence), which supported us to continue treatment of mycoplasma infection. On the fourth day after admission, the tracheal tube was removed and HFNC was used to assist ventilation. On the fifth day after admission, the patient's CRP returned to normal (7.2 mg/L), meropenem was discontinued and replaced with cefoperazone sodium and sulbactam sodium. However, the detection of fibrinogen was 1.76 g/L, D-dimer was significantly increased [>40 mg/L, fibrinogen equivalent units (FEU)], and we added a small dose of low molecular weight heparin to anticoagulant therapy. During the course of the disease, the bone marrow smear test revealed increased hemophagocytic macrophages, and the highest serum ferritin was 5,861.4 μg/L and the highest LDH was 2922 U/L (normal, 120–246 U/L). Cytokines were increased (soluble interleukin-2 receptor, IL-2, 2,888.1 U/ml, IL-8 46.2 pg/ml, IL-10 41.68 pg/ml, TNF-α 25.71 pg/ml). The patient had an H score of 236, with a 98%–99% probability of HLH (H score >169 had a sensitivity of 93% and specificity of 86% for the diagnosis of HLH) (7). Tests for Epstein–Barr virus (EBV) and cytomegalovirus (CMV) were negative. Therefore, we reconfirmed the consideration that HLH was secondary to MP and continued glucocorticoid therapy. On the 11th day after admission, the patient's body temperature was normal for 8 days, the CT lung window showed the lesions in both lungs were significantly reduced and faded (Figure 1B), and the patient was discharged. After 1 month of regular oral prednisone, she did not experience any more of those initial symptoms. At the 4-month follow-up, all indicators remained normal. The timeline of disease progression and treatment is summarized in Figure 2.

## System review

A review of the literature between 1998 and 2024 was undertaken using databases such as PubMed, Web of Science, Embase, and Medline. The search employed the keywords “diffuse alveolar hemorrhage,” “hemophagocytic syndrome/hemophagocytic lymphohistiocytosis,” “diffuse alveolar hemorrhage,” “Mycoplasma pneumonia,” and “Children.” The relevant information was extracted from the selected articles, including the first author’s name, year of publication, country of study, age range of patients, underlying disease, etiology, clinical manifestation, relevant examination, treatment, hospital length of stay, in-hospital mortality, cause of death, and follow-up time. In Table 1, we summarize the data of one published article, which revealed in detail the treatment of pediatric patients with MP-associated DAH (6). Seven published articles are summarized in Table 2, focusing on pediatric patients with HLH secondary to MP (1, 2, 4, 8–11).

**Table 1:** Published articles on pediatric cases of diffuse alveolar hemorrhage induced by *Mycoplasma pneumoniae* infection.

Author	Country, year	Male/female	Age	Chief complaint	Underlying disease	Other complications	Etiology	Pathogen/specimen/method	Diagnostic method	Hospital length of stay at diagnosis	Treatment method	Hospital length of stay	In hospital mortality	Follow-up time
Xinjuan Zhang et al. (6)	China, 2022	0/1	8 years	Cough for 1week, fever for 4 days, and dyspnea for 5 h	None	ARDS	Infection	*Mycoplasma pneumoniae*/pleural fluid and blood/mNGS	BAL	48 h	Azithromycin (3days × 2) and other antibiotics, Human blood immunoglobulin, epinephrine endotracheal instillation, glucocorticoids (1–2 mg/kg d, ivgtt, 14 days), fresh frozen plasma and fibrinogen infusions Mechanical ventilation after 48 h admission for 10days VV-ECMO after 3 days admission for 5 days	17 days	0.0%	45 days

ARDS, acute respiratory distress syndrome; mNGS, metagenomic next-generation sequencing; BAL, bronchoscopy and bronchoalveolar lavage; VV-ECMO, veno-venous extracorporeal membrane oxygenation; IPH, idiopathic pulmonary hemosiderosis; CT, computed tomography.

**Table 2:** Published articles on pediatric cases of hemophagocytic lymphohistiocytosis induced by *Mycoplasma pneumoniae* infection.

Author	Country, year	Male/female	Age	Clinical manifestation	Underlying disease	Other complications	Etiology	Pathogen/specimen/method	WBC (/μl)	HB (g/dl)	PLT (/μl)	Serum C-reactive protein (mg/L)	AST (IU/L)	ALT (IU/L)	LDH (IU/L)	TG (mg/dl)	Ferritin (ng/ml)	IL-2R (U/ml)	Fibrinogen (mg/dl)	Bone marrow aspiration	Course at diagnosis (days)	Treatment method	Hospital length of stay	In hospital mortality (%)	Cause of death	Follow-up time
Yasushi Ishida et al. (8)	Japan, 2004	1/1	11 years, 10 years	Fever (2), cough (2), and dyspnea (1)	None	None	Infection	MP/pharyngeal swab/passive agglutination test	5,500; 3,500	13.1, 14.4	136,000–193,000	10.7, 3.8	484; 1,109	191; 866	3,327; 1,640	Undisclosed	19,620; 2,553	2,080; 2,056	Undisclosed	Hemophagocytic macrophages increased	11 and 6	CAM (1), CTM (1), EM (1), AZT (1); CFTM-PI, (1); MINO (2), prednisolone (2)	21 days, 11 days	0.0	None	10 years, 3 years
Megumi Yoshiyama et al. (1)	Japan, 2008	2/2	1–11 years	Fever (4), Hepatomegaly (2), splenomegaly (1)	Undisclosed	Consciousness disturbance, paralytic ileus	Infection	MP/serial serum samples/particle agglutination method	3,800–8,300	7.7–13.7	57,000–131,000	0.14–10.9	Undisclosed	26–67	508–4,236	Undisclosed	1,070–36,050	3,002; >3,200 (2, none)	Undisclosed	Hemophagocytic macrophages increased	7–11	CAM (4),EM (4), MINO (4) IVIG (2), glucocorticoids (1)	Undisclosed	0.0	None	Undisclosed
Bruch LA et al. (9)	The USA, 2001	1/0	12 years	Cough, headache, photophobia, nausea, and vomiting	None	Meningoencephalitis	Infection	MP/serial serum samples/particle agglutination method	25,700	12.9	551,000	Undisclosed	Undisclosed	Undisclosed	Undisclosed	Undisclosed	Undisclosed	Undisclosed	Undisclosed	Undisclosed	7s	Undisclosed	60 h	100.0	Brain death	None
Yuji Koike et al. (2)	Japan, 2013	0/1	7 years	Fever, cough, and malaise	None	None	Infection	MP/serum/passive agglutination test	1,200	11.8	<10,000	6.27	48	28	921	434	2,251	Undisclosed	Undisclosed	None	4	Clarithromycin, prednisolone	11 days	0.0	None	More than 3 years
Lu Zhi-wei et al. (4)	China, 2014	2/1	1, 3, 6 years	Fever (3), cough (3), coma (1)	Undisclosed	Undisclosed	Infection	MP/BAL, pharyngeal swab/passive agglutination test and molecular diagnostic testing (3)	200–1,600	7.9–10.3	64,000–157,000	Undisclosed	Undisclosed	Undisclosed	1,170–1,285	Undisclosed	936.7–7,477	Undisclosed	130–180	Hemophagocytic macrophages increased (3)	2–30	AZT combined with other antibiotics (3), mechanical ventilation (2), IVIG (3), glucocorticoids (3), CyA + VP-16 (1) Fiber optic bronchoscope (3)	47, 20, 25 days	33.3	Intracranial hemorrhage	Undisclosed
Motoko Yasutomi, et al. (10)	Japan, 2016	1/0	3 years	Fever and cough	None	None	Infection	MP/serum/passive agglutination test	4,800	11.9	116,000	59.4 (normal <3.2)	1,788	332	3,748	Undisclosed	7,718 (normal, 18.6–261)	2,686 (normal, 144.5–518)	143 (normal, 140–340)	Hemophagocytic macrophages increased	4	CAM, ABPC/SBT, MEPM, and MINO, IVIG, 2 g/kg prednisolone, 2.6 mg/kg/d, 13 days	Undisclosed	Undisclosed	Undisclosed	Undisclosed
Gu Jia-li et al. (11)	China, 2020	6/5	7 months–9.5 years	Fever (11), cough (8), hepatomegaly (11)	None	Undisclosed	Infection	MP/BAL, pharyngeal swab/passive agglutination test (7) and molecular diagnostic testing (10)	Undisclosed	7.7 (median)	45,000 (median)	Undisclosed	Undisclosed	Undisclosed	1,285 (median)	4.64 (median) (9)	7,260 (median)	Undisclosed	126 (median) (6)	Hemophagocytic macrophages increased (11)	6–30	AZT combined with other antibiotics (11), mechanical ventilation (5), IVIG (10), glucocorticoids (10), CyA (6) + VP-16 (1), Fiber optic bronchoscope (7)	Undisclosed	18.2	Multiple organ failure	Undisclosed

MP, *Mycoplasma pneumoniae*; WBC, white blood cell count; HB, hemoglobin; PLT, platelets; AST, aspartate aminotransferase; ALT, alanine aminotransferase; LDH, lactate dehydrogenase; TG, triglyceride; IL-2R, soluble interleukin-2 receptor; CTM, cefotiam; EM, erythromycin; AZT, azithromycin; CFTM-PI, cefteram pivoxil; IVIG, intravenous immunoglobulin; CAM, clarithromycin; ABPC/SBT, ampicillin/sulbactam; MEPM, meropenem; MINO, minocycline; BAL, bronchoscopy and bronchoalveolar lavage; CyA, cyclosporine A; VP-16, etoposide.

.

## Discussion

Currently, after the quarantine-related measures were lifted, there was a surge of endemic MP infections in children in countries such as the United States, Switzerland, Sweden, England, Slovenia, and China (12). MP is the most important pathogen of community-acquired pneumonia in children aged over 5 years in China and has even become one of the most important pathogens of respiratory infections (13, 14). As a pathogen without a cell wall, the infection rate of MP has again increased rapidly (15), which may affect the population who have not been exposed to MP in the past 3 years through the two main pathogenesis mechanisms of direct pathogen injury and abnormal host immune response, and lead to rare severe intra- and extrapulmonary manifestations (16). The most common intra- and extrapulmonary manifestations were plastic bronchitis, pulmonary embolism, necrotizing pneumonia, and acute attack of asthma. The main extrapulmonary manifestations were nervous system involvement and skin mucosal damage (14). Both DAH as an intrapulmonary manifestation of MP infection and/or HLH as an extrapulmonary manifestation are very rare in children. Children with infection-associated DAH have high mortality in the acute phase (4, 5, 11, 17). DAH is a clinical syndrome with a wide range of causes. Children with DAH often have complex, critical conditions and rapid progression, which can lead to rapid respiratory failure. The mortality rate of DAH in the acute phase was high, up to 75%, especially for DAH caused by infection (5, 17).

Typical clinical manifestations of DAH include hemoptysis (67%), anemia, and hypoxic respiratory failure (5, 18). Before the deadline, only one case of DAH secondary to MP infection has been reported in children (6). That patient was admitted with hypoxic respiratory failure. At 48 h after admission, she showed the above three symptoms and was treated with invasive ventilator respiratory support and veno-venous extracorporeal membrane oxygenation (VV-ECMO). During the course of the disease, she acquired a fungal infection. She was discharged successfully after 17 days in hospital and no abnormality was found after 45 days of follow-up. In our case, the child was also admitted with hypoxic respiratory failure. When dyspnea was not significantly relieved on the day of admission, the patient was changed to invasive ventilator respiratory support, and the time of tracheal intubation was not more than 4 days. There was no fungal infection during the course of the disease, and the patient was discharged after 10 days of hospitalization. The follow-up period was up to 4 months. This case may help identify atypical clinical manifestations of MP-DAH for timely recognition and management. Children with MP-DAH only showed hypoxic respiratory failure, which may be related to the developmental characteristics of children with incomplete cough reflex and respiratory muscle development and high airway resistance (half of the children had no symptoms of hemoptysis) (18). We believe that the absence of hemoptysis and anemia cannot be used as a basis for excluding DAH. The review published by Reisman et al. (18) also clearly suggests that chest X-rays in patients with DAH usually show alveolar opacity, CT may indicate the extent of the disease, and chest imaging may be used to assist in the diagnosis of DAH. Therefore, we consider that patients presenting only with hypoxic respiratory failure and significantly decreased Hb in the absence of bleeding elsewhere can be combined with chest imaging to assist the diagnosis of DAH.

At present, the etiological diagnosis of DAH is mostly based on clinical diagnosis or tissue biopsy or genetic testing. Common clinical etiologies in children include related complications or sequelae after infection, immune diseases, cardiovascular disease, and airway lesions, and are exemplary in the diagnosis of idiopathic pulmonary hemosiderosis (IPH) syndrome, etc. (5, 19). In this case, the child had definite MP infection. Despite the high sensitivity and specificity of the MP nucleic acid test (13), the nucleic acid test for MP was negative on day 3 of the course, but the antibody detection and mNGS test for MP were positive on day 6 of the course, which suggested that the early negative MP test should not exclude the pathogen. Once a pathogen infection was suspected, repeated and multiple methods of detection can be considered to search for pathogens. mNGS detection was helpful for patients with DAH to search and identify pathogens relatively comprehensively. Unfortunately, we did not perform autoantibody and other immune screening and bronchoscopy. Although the children were followed up for 4 months and glucocorticoids were discontinued for more than 3 months, no initial symptoms recurred and all indexes were normal. We considered it unlikely that this patient had an immune-related disease. We will continue to dynamically track the follow-up symptoms, signs, and indicators in this child, and consider immune-related screening after full communication and consultation with the families when necessary.

It has been reported in many countries that MP is recognized to activate B and T lymphocytes, as well as cytokines secreted by various cells, which initiates the immune cascade and causes MP-HLH (4). Of the 23 reported cases (1, 2, 4, 8–11) of MP-HLH (1 case with incomplete information), 8 cases were diagnosed after 2 weeks of the course of the disease, with a mortality rate of 37.5% (3/8 cases), and 14 cases were diagnosed within 2 weeks of the course of the disease, with a mortality rate of 7.1% (1/15 cases). Two patients died of meningoencephalitis and intracranial hemorrhage, and the other two cases both died of multiple organ failure. MP-HLH has acute onset, rapid progression, and high mortality, which may be related to late diagnosis or other serious complications (4). HLH may also be overlooked because of its clinical similarity to exacerbations such as autoimmune diseases or sepsis. For instance, patients with inflammatory bowel disease (IBD) infected with CMV, EBV, and other viruses may be considered as IBD exacerbations while HLH may be ignored, resulting in a misdiagnosis or delayed diagnosis, leading to a poor prognosis (20). In particular, the clinical features and laboratory findings of HLH often overlap with those of sepsis-related multiple organ failure, which may lead to a delayed diagnosis and treatment. The H score is helpful to distinguish HLH from sepsis-related multiple organ failure and can assist in the diagnosis of secondary HLH (21). In this case, the patient had an H score of 236, which supported the diagnosis of secondary HLH (probability 98%–99%), and treatment was initiated on the second day after admission and the patient was discharged successfully. The early identification of MP-HLH may help reduce mortality. EBV and CMV are the most common pathogens of secondary HLH in children, but diagnosis and treatment may be delayed due to omission of other pathogens. For example, the clinical manifestations of HLH induced by CMV infection were similar to those of COVID-19 infection, and HLH should be considered when fever and abnormal liver function are observed (22). The mNGS test results of the patient in this case confirmed MP infection, and ruled out the possibility of EBV, CMV, COVID-19, and other pathogen agents causing HLH. It has also been proposed (1, 2) that although MP-HLH is rare, healthcare professionals should be highly vigilant of HLH in refractory *Mycoplasma* infections with cytopenia. In this case, the child’s MP was not difficult to treat, and two courses of azithromycin treatment were effective. Therefore, we consider that professionals should be vigilant for MP combined with HLH when MP infection is complicated with cytopenia, regardless of whether MP is refractory or not.

## Conclusion

The child in this case had HLH on admission, and manifestations of DAH were quickly recognized. Although the MP nucleic acid test was negative, we still highly suspected MP infection by epidemiology (epidemic year, season, age, cough manifestation), empirically selected azithromycin to treat MP infection, and confirmed MP infection with a further antibody test and mNGS. Combined with a retrospective review and comprehensive analysis of several related reports, we concluded the following: (1) mNGS may become an important method for the detection of pathogens for DAH and/or HLH; (2) the clinical manifestations of DAH in children may not be typical, but only acute hypoxic respiratory failure and Hb decreased significantly without bleeding elsewhere, a combination of chest imaging can be considered to assist the diagnosis; and (3) when MP infection is complicated with cytopenia, whether MP is refractory or not, MP complicated with HLH should be considered.

## Data Availability

The datasets presented in this study can be found in online repositories. The names of the repository/repositories and accession number(s) can be found in the article/Supplementary Material.
